# Prevalence of heavy episodic drinking and associated factors among adults residing in Arba Minch health and demographic surveillance site: a cross sectional study

**DOI:** 10.1186/s12889-020-09998-3

**Published:** 2020-12-09

**Authors:** Befikadu Tariku Gutema, Adefris Chuka, Gistane Ayele, Eshetu Zerhun Tariku, Zeleke Aschalew, Alazar Baharu, Nega Degefa, Mekdes Kondale Gurara

**Affiliations:** 1 School of Public Health, Arba Minch University, P.O.Box 21, Arba Minch, Ethiopia; 2 Arba Minch Health and Demographic Surveillance System (HDSS), Arba Minch, Ethiopia; 3 CARE Ethiopia Hawassa Project Office, Hawassa, Ethiopia; 4 School of Nursing, Arba Minch University, Arba Minch, Ethiopia; 5 Department of Computer Science, Arba Minch University, Arba Minch, Ethiopia

**Keywords:** Alcohol, Heavy episodic drinking, Adults, Health and demographic surveillance system

## Abstract

**Background:**

Alcohol consumption is associated with different types of illnesses; particularly heavy episodic drinking is one of the risk factors for the disease burden of alcohol intake. The aim of the study was to assess the prevalence of heavy episodic drinking and associated factors in Arba Minch Health and Demographic Surveillance Site (HDSS).

**Methods:**

A community-based cross-sectional study was conducted in 2017 among adult residents of Arba Minch HDSS. Using Arba Minch HDSS database, 3368 individuals were selected by simple random sampling techniques. From WHO STEPS instruments, step one was applied for this study. Variables with a *p*-value of less than 0.10 for bivariate analysis entered into a multivariable logistic regression model to outline the independent predictors of the heavy episodic drinking. To assess the presence of an association between dependent and independent variables, a *p*-value of less than 0.05 was considered.

**Results:**

The prevalence of heavy episodic drinking was 13.7% (95% CI: 12.6–14.9). The study has shown that heavy episodic drinking was significantly associated with occupation (daily laborer [AOR = 0.49; 95% C.I: 0.29–0.85] and housewives [AOR = 0.63; 95% C.I: 0.45–0.88] compared with farmers), wealth index (2nd quintiles [AOR =0.55; 95% C.I: 0.41–0.74) and 3rd quintiles [AOR = 0.66; 95% C.I: 0.46–0.93] compared with 1st quintiles), and climatic zone (midland [AOR = 1.80;95% CI: 1.11–2.93), highland [AOR = 1.95;95% CI: 1.19–3.18] compared with lowland). In addition, tobacco use [AOR = 4.28;95% CI: 3.38–5.43], and khat use [AOR = 4.75; 95% CI: 2.66–8.50) were also associated with heavy episodic drinking among the study participants.

**Conclusions:**

More than one in ten adults reported heavy episodic drinking in the study area. Intervention programs that aim to prevent heavy episodic drinking should be designed appropriately for individuals from lower wealth status, and for highlander.

## Background

Heavy episodic drinking is associated with different types of illnesses including cardiovascular disorders, different forms of cancer, liver cirrhosis, and chronic pancreatitis [1–5]. Harmful use of alcohol contributes to the development of mental and behavioral disorders [6]. In addition to its contribution to morbidity and mortality due to non-communicable disease (NCD), alcohol contributes to the mortality due to infectious diseases, injuries, and violence [1, 7–9]. Alcohol is also related to the decrease in productivity, the increase in the incidence of disability and crime, for the poor academic performance and the exposure for traffic accidents [10–14]. In general, alcohol consumption is one of the major risk factors for the burden of diseases [13]. Heavy episodic drinking is one of the risk factors for the disease burden of alcohol intake [6, 9, 13, 15].

In 2016, the global current drinkers were reported to be 43.0% and for Africans, it was 32.2% among age above 15 years [7]. Ethiopia Demographic and Health Survey (EDHS) 2016 reported that 35% of women and 46% of men reported drinking alcohol at some point in their lives [16]. The global prevalence of heavy episodic drinking, which is defined as consumption of 60 or more grams of pure alcohol drinks on a single occasion at least once per month, was 18.2% in 2016. In this report, heavy episodic drinking was 9.3% among Ethiopia age above 15 years [7].

World Health Organization (WHO) recommended both the prevention and treatment of harmful use of alcohol as a strategy for reducing its public health impact [17]. For the Ethiopian population above 15 years in 2016, the total pure alcohol per capita consumption, total consumption, and average daily intake were 2.8 l, 12.6 l, and 27.2 g, respectively [7]. In Ethiopia, drinking habits have been changing and the number of alcohol beverage manufacturers has been increasing, which indicates this situation as alarming for increased alcohol intake [18]. With the vision of a world free of the avoidable burden of NCDs, the WHO recommended a 10% relative reduction in the harmful use of alcohol in 2025 [19]. On top of this, the United Nations Sustainable Development Goals described strengthening the prevention and treatment of harmful use of alcohol and other substance use among adults [20]. The Ethiopian parliament passed a bill that bans alcohol advertisements through mainstream media and increased the legal age limit for alcohol to 21 years of age (Proclamation No.1112/2019). To see the effectiveness of programs and the attainment of the goals, assessing the magnitude of excess alcohol intake is mandatory. Thus, the aim of the study was to assess the prevalence of heavy episodic drinking and associated factors in the Arba Minch Health and Demographic Surveillance Site (HDSS), which was collected based on the WHO STEPwise approach to Surveillance (STEPS).

## Methods

A community-based cross-sectional study was conducted from April to June 2017 in Arba Minch HDSS. Arba Minch HDSS includes nine Kebeles of Arba Minch Zuria District, Southern Ethiopia. Adult residents (25–64 years) of Arba Minch HDSS were considered as a source population. Pregnant mothers and women who have history of recent delivery up to 8 weeks were excluded from the study. WHO STEPS guideline was followed for calculating the sample size and it was 3368. The sampling frame was obtained from Arba Minch HDSS and a simple random sampling technique was implemented to select the study participants. For the extraction of the sampling frame, sex, date of birth, and individual and household identifications were used. The sampling frame was stratified in to eight groups (based on sex and age categories) and STATA version 14 was used for selecting the study participants from each stratum. The WHO STEPS instruments and data collection procedures were adapted for data collection. Step one of the STEPS approach was applied for this study, which depends on the questionnaire. The questionnaire was designed to obtain core data on socio-demographic information, tobacco and alcohol use, and physical activity [21]. Variables for wealth index**,** khat chewing, and mental stress were included in the questionnaire, which are not a part of the STEPS guideline. Questionnaire from Ethiopia Demographic and Health Survey (EDHS), which was based on the household ownership of productive asset and household characteristics, were used to categorized wealth status of the participants [16]. Self-Reporting Questionnaire (SRQ-20) was used for examining the mental stress status of the participants [22]. The interview guides were described elsewhere [16, 21, 22]. The interview was conducted at the participants’ house and alone. Data collectors and supervisors of Arba Minch HDSS were trained for three days on data collection material. Modifications were made on the tools based on the identified gaps from the pre-test which was conducted on 2% of the sample size. Monitoring the data collection process was done by supervisors. In addition, they checked the data for completeness every day during the data collection time. The data collectors repeatedly visited (at least three times) the participants who were absent during data the initial collection time. Details of its methods have been described elsewhere [23].

EPIData version 3.1 statistical software was used for data entry (single data entry) and the data were exported to STATA version 14 for data management and analysis. Wealth index was computed using a principal component factor analysis and grouped into five quantiles. Variables from the STEPS tool was generated in accordance with the manual [21]. Altitude below 1800, 1800–2400 and above 2400 m above sea level classified as lowland, midland and highland, respectively [24]. Physical activity levels with average metabolic equivalents-minutes per week < 600, 600–3000 and > 3000 were grouped into low, moderate and high, respectively [21]. Based on the self-reporting questionnaire (SRQ-20), mental stress was categorized into three (mild, moderate, and severe) [22]. Heavy Episodic Drinking or Excessive Alcohol Consumption in this study is defined as the consumption of 6 or more drinks for men and 4 and more drinks for women on a single occasion at least once per month [7]. A binary logistic regression model was employed to assess the possible association of the independent and dependent variables. Variables with a *p*-value of less than 0.10 in bivariate analysis and variables with significant importance were entered into a multivariable logistic regression model to outline the independent predictors of the heavy episodic drinking. To assess the presence of an association between dependent and independent variables, a *p*-value of less than 0.05 in the final model was considered. For the assessment of multicollinearity, variable inflation factors were used and non-collinear variables were used. The Hosmer-Lemeshow test was checked to assess the goodness-of-fit model and the *P*-value was 0.515.

## Results

### Socio-demographic characteristics of the study participants

A total of 3346 adults have participated in the study, making a response rate of 99.3%. The mean (SD) age of the study participants was 44.6(11.2) years. Half of the participants were female (50.0%) and farmers (53.2%). Majority of the participants had no formal education (69.8%), married (87.9%), from Gamo ethnic background (81.1%) and rural residents (83.7%). A significantly higher proportion of heavy episodic drinking was observed among the Orthodox religious followers (Table 1).

**Table 1:** Socio-demographic characteristics of the study participants (*N* = 3346) by heavy episodic drinking, Arba Minch HDSS, South Ethiopia, 2017

Characteristics	Categories	Heavy Episodic Drinking		Total Freq. (%)
		No Freq. (%)	Yes Freq. (%)	
Sex	Male	1409 (84.17)	265 (15.83)	1674 (50.03)
	Female	1479 (88.46)	193 (11.54)	1672 (49.97)
Age group	25–34	712 (88.56)	92 (11.44)	804 (24.03)
	35–44	736 (85.28)	127 (14.72)	863 (25.79)
	45–54	732 (86.63)	113 (13.37)	845 (25.25)
	55–64	708 (84.89)	126 (15.11)	834 (24.93)
Ethnicity	Gamo	2331 (85.92)	382 (14.08)	2713 (81.08)
	Zeyse	251 (86.55)	39 (13.45)	290 (8.67)
	Other	306 (89.21)	37 (10.79)	343 (10.25)
Religion	Protestant	2037 (96.82)	67 (3.18)	2104 (62.88)
	Orthodox	718 (68.12)	336 (31.88)	1054 (31.50)
	Other	133 (70.74)	55 (29.26)	188 (5.62)
Marital status	Never married	105 (90.52)	11 (9.48)	116 (3.47)
	Married	2516 (85.55)	425 (14.45)	2941 (87.90)
	Divorced	14 (82.35)	3 (17.65)	17 (0.51)
	Widowed	219 (93.59)	15 (6.41)	234 (6.99)
	Separated	34 (89.47)	4 (10.53)	38 (1.14)
Occupation	Farmer	1459 (82.01)	320 (17.99)	1779 (53.17)
	Daily laborer	237 (92.22)	20 (7.78)	257 (7.68)
	Merchant	130 (89.04)	16 (10.96)	146 (4.36)
	House wife	838 (91.68)	76 (8.32)	914 (27.32)
	Employed at Org.	93 (87.74)	13 (12.26)	106 (3.17)
	Other	131 (90.97)	13 (9.03)	144 (4.30)
Educational status	No formal educe	1992 (85.35)	342 (14.65)	2334 (69.75)
	Primary	680 (88.54)	88 (11.46)	768 (22.95)
	Secondary & above	216 (88.52)	28 (11.48)	244 (7.29)
Wealth index	1st quantile	512 (76.42)	158 (23.58)	670 (20.02)
	2nd quantile	575 (85.69)	96 (14.31)	671 (20.05)
	3rd quantile	587 (88.01)	80 (11.99)	667 (19.93)
	4th quantile	609 (91.03)	60 (8.97)	669 (19.99)
	5th quantile	605 (90.43)	64 (9.57)	669 (19.99)
Residency	Rural	2380 (84.97)	421 (15.03)	2801 (83.71)
	Urban	508 (93.21)	37 (6.79)	545 (16.29)
Climatic zone	Lowland	1385 (91.72)	125 (8.28)	1510 (45.13)
	Midland	369 (86.42)	58 (13.58)	427 (12.76)
	Highland	1134 (80.48)	275 (19.52)	1409 (42.11)

### Lifestyle related characteristics of the study participants

Nearly 20% of the study participants were current tobacco consumers. Most (64.3%) of the study participants involved in high physical activity. Nearly 2% of the participants chew khat and 4% had severe mental stress levels (Table 2).

**Table 2:** Lifestyle related characteristics of the study participants (N = 3346) by heavy episodic drinking, Arba Minch HDSS, South Ethiopia, 2017

Characteristics	Categories	Heavy Episodic Drinking		Total Freq. (%)
		No Freq. (%)	Yes Freq. (%)	
Current tobacco use	No	2433 (91.16)	236 (8.84)	2669 (79.77)
	Yes	455 (67.21)	222 (32.79)	677 (20.23)
Physical activity level	Low	715 (90.39)	76 (9.61)	791 (23.64)
	Moderate	365 (90.12)	40 (9.88)	405 (12.10)
	High	1808 (84.09)	342 (15.91)	2150 (64.26)
Khat chewing	No	2846 (86.77)	434 (13.23)	3280 (98.03)
	Yes	42 (63.64)	24 (36.36)	66 (1.97)
Mental stress level	Mild	1907 (85.94)	312 (14.06)	2219 (66.32)
	Moderate	870 (87.44)	125 (12.56)	995 (29.74)
	Severe	111 (84.09)	21 (15.91)	132 (3.95)

### Alcohol consumption

Nearly 40% (95% CI: 38.6–41.9) of the participants reported that they have consumed alcohol in their lifetime; of whom 80.1% (1079) have consumed alcohol in the past 12 months. This means that from the total participants, 32.3% (95% CI: 30.7–33.9) took alcohol within the past 12 months. From those who took alcohol last year, 14.0% (151), 39.5% (426) and 46.5% (502) took alcohol at least daily, at least once per week and at least once per-month, respectively.

### Prevalence of heavy episodic drinking

From the total participants, 27.4% (95% CI: 25.1–29.9) had consumed alcohol within the past 30 days. Nine hundred study participants recalled the frequency of having alcohol at least one standard drink in the past 30 days and the mean (SD) was 3.9(3.3). The prevalence of heavy episodic drinking was 13.7% (95% CI: 12.6–14.9).

### Factors associated with heavy episodic drinking

Based on bivariate analysis, the odds of heavy episodic drinking were significantly higher among males compared to females, and older age groups especially 35–44 and 55–64 compared to 25–34 years old. In addition, significantly lower odds were observed among those who attended higher education especially primary education compared to no formal education, and urban residents compared to rural.

Based on multivariable analysis, the odds of heavy episodic drinking were significantly lower among daily laborer (AOR 0.49, 95% CI: 0.29–0.85) and housewives (AOR 0.63, 95% CI: 0.45–0.88) compared to the farmer. The probability of having heavy episodic drinking was lower among participants living in higher wealth quantile households. Significantly lower odds of heavy episodic drinking were observed among 2nd and 3rd quantile, which was nearly by half for 2nd quantile (AOR 0.55, 95% CI: 0.41–0.74) and one third for 3rd quantile (AOR 0.66, 95% CI: 0.46–0.93). For those who live at midland (AOR 1.80, 95% CI: 1.11–2.93) and highland (AOR 1.95, 95% CI: 1.19–3.18), the probability of having heavy episodic drinking increased compared with lowlands. Among current tobacco users and Khat chewers, the probability of consuming heavy episodic drinking was more than four times (AOR is 4.28 [95% CI: 3.38–5.43] and 4.75 [95% CI: 2.66–8.50], respectively) (Table 3).

**Table 3:** Multivariable logistic regression analysis of factors associated with heavy episodic drinking among study participants (N = 3346), Arba Minch HDSS, South Ethiopia, 2017

Characteristics	Category	COR	AOR (95% CI)
Sex	Male	1	
	Female	0.69**	1.12 (0.86, 1.45)
Age group	25–34	1	
	35–44	1.34*	1.05 (0.77, 1.44)
	45–54	1.19	0.83 (0.6, 1.16)
	55–64	1.38*	0.82 (0.58, 1.15)
Occupation	Farmer	1	
	Daily laborer	0.38**	0.49* (0.29, 0.85)
	Merchant	0.56*	0.88 (0.48, 1.59)
	Housewives	0.41**	0.63** (0.45, 0.88)
	Employed at Org.	0.64	0.9 (0.45, 1.77)
	Other	0.45*	0.62 (0.33, 1.17)
Educational status	No formal education	1	
	Primary	0.75*	1.23 (0.9, 1.7)
	Secondary & above	0.76	1.27 (0.74, 2.17)
Wealth index	1st quantile	1	
	2nd quantile	0.54**	0.55** (0.41, 0.74)
	3rd quantile	0.44**	0.66* (0.46, 0.93)
	4th quantile	0.32**	0.94 (0.56, 1.57)
	5th quantile	0.34**	0.97 (0.54, 1.73)
Residency	Rural	1	
	Urban	0.41**	0.7 (0.46, 1.06)
Climatic zone	Lowland	1	
	Midland	1.74*	1.8* (1.11, 2.93)
	Highland	2.69**	1.95** (1.19, 3.18)
Current tobacco use	No	1	
	Yes	5.03**	4.28** (3.38, 5.43)
Khat chewing	No	1	
	Yes	3.75**	4.75** (2.66, 8.5)

COR: Crud Odds Ratio; AOR: Adjusted Odds Ratio; CI: Confidence Interval; * *P*-value < 0.05; ** *P*-value < 0.01; Div/Wido/Seprt: Divorce, Widowed and Separated

## Discussion

The prevalence of heavy episodic drinking was 13.7% among study participants. A national WHO STEPS based survey for Ethiopian and Kenyan showed that the prevalence of heavy episodic drinking were 12.4 and 12.7%, respectively [25, 26], which are similar to this finding. WHO report indicated that the prevalence of heavy episodic drinking was 9.7% for the Ethiopian population 15 years and older. That report also showed that the prevalence were 18.2% for the global and 17.4% for the African. This indicates that the prevalence of heavy episodic drinking was higher than the WHO report for the country [7]. The finding showed that there is a need to work very hard to achieve the goal for the area in 2025 (a 10% relative reduction of heavy episodic drinkers) [19].

This study has found that nearly 60% of adults had never consumed alcohol. The national report of Ethiopian showed that half of the population of Ethiopia had a lifetime abstain from taking alcohol [25]. In 2016, the WHO reported that 45% of the global population never consumed alcohol [7]. In this study, around 32% of the study participants were current drinkers (took alcohol in the past 12 months). A systematic review among university students in Ethiopia reported that the current use of alcohol was 26.65%, [27], which is lower than this finding. The national report showed that nearly 40% of Ethiopian adults age 18–64 years took alcohol one year preceding the survey [25]. According to the WHO report in 2016, 43.0% of the world population and 32.2% of the African population were current drinkers [7]. With the consideration of the WHO 2016 report, the finding of this result is nearly similar to the African countries. The drinking habits of Ethiopians have been changing. Currently, it is common to use bars as meeting points during working hours for most of the young men especially working in informal sectors, and having alcohol at the time of breakfast (morning) for those whose employment does not require strong supervision [18]. The finding of this report on the proportion of current drinker and heavy episodic drinking indicate the need for significant effort for attaining the goals of WHO, which is a 10% reduction in 2025 [19].

Even if not significantly associated with multivariable logistic regression analysis, based on bivariate analysis, the odds of heavy episodic drinking were significantly higher among males, older age groups, low educational attainment, and rural residents. Similar to this finding of bivariate analysis, the STEPS survey for Ethiopian and Kenyan indicated that male and rural residents were more likely to involve in heavy episodic drinking [25, 26]. EDHS 2016 report also showed that alcohol consumption is increases with age [16].

The difference in the prevalence of heavy episodic drinking among different occupation groups might be related to the availability of alcohol, customs of the member of the group related to the same occupation, or characteristics of certain jobs like job variability, supervision, and qualification [28]. In this study, the likelihood of being heavy episodic drinking was lower among housewives and daily laborer compared to the farmer. An analysis of health survey for England (2003) showed that those involved in skilled non-manual, skilled manual and partially/unskilled manual occupational groups were involved significantly in heavy episodic drinking compared to professional’s occupational groups [29]. A report based on the analysis of Korea National Health and Nutrition Examination Survey also showed that clerical support workers, service and sales workers were higher mean alcohol use disorders identification test score [30].

The likelihood of heavy episodic drinking decreased as wealth status increased, with a significant decrease among second and third quantile groups compared to the first. Different studies also showed that heavy consumption of alcohol is associated with lower socioeconomic status [31, 32]. Even if the study was conducted among adolescents, the report from New Zealand showed that lower socioeconomic groups were at higher risk for consumption of alcohol [33]. A study about the socioeconomic status as an effect modifier of alcohol consumption and harm based on a linked cohort data of the Scottish Health Surveys indicated that alcohol-attributable harms, including binge drinking, were higher among disadvantaged social groups [34]. A study on stress, social support and the problem of drinking among women in poverty based on a welfare client longitudinal study in America indicated that women in poverty are exposed to stressors that increase the problem of drinking [35].

In this study, the odds of heavy episodic drinking were significantly higher among those who live in midland and highland compared to lowland. As altitude increases the temperature decrease [36], which may contribute to an increase in the likelihood of heavy episodic drinking among midlands and highland residents.

Heavy episodic drinking was more common among the study participants who were current tobacco users than their counterparts. Different studies showed that alcohol and tobacco use are highly correlated behaviors and those who drink are very likely to smoke and vice versa [37–40]. Based on Shiffman & Balabanis (1995), the phenomena used to explain for the association of alcohol and tobacco were both the between-person and the situation covariation. The between-persons is explained by the use of one drug that is likely to use the other, which means the use of heavy episodic drink of alcohol increase the probability of smoking and vice versa. Regarding the situation covariation, which indicated that the two drugs tend to be used together, for instance in time of stress [41].

Khat (*Catha edulis*) is a chewable fresh-leave used as stimulant commonly in East Africa. It contains amphetamine-like stimulatory, which is a closely similar effect with cathinone [42, 43]. In this finding, those participants who chew Khat were more likely to involve in heavy episodic drinking compare to non-chewers. Different studies found a similar relationship, which indicates that both uses of khat chewing and heavy episodic drinking are associated [44–47]. A study among Ethiopian Universities indicated that the three substances used in combination were khat, alcohol, and cigarettes [44]. Khat chewers use to stay awake, increase productivity or feel ‘high’. Alcohol is needed to rest, calm their nerves and sleep, which commonly used to counteract the stimulating effect of khat [48].

Even if the study used the STEPS guideline, which makes it easy to compare with studies conducted based on a similar method and definition of heavy episode drinking, there are limitations. The data were collected using interview including alcohol intake. This self-reported information regarding alcohol intake use may be subjected to recall errors. In addition, it is culturally unacceptable to openly acknowledge drinking habits for unemployed youth and housewives. Most of the alcoholic beverages were made locally, which does not have a standard alcoholic content and with a significant difference in content to estimate the standards [49, 50]. Because of the cross-sectional nature of the study, it is difficult to determine which comes first in variables like heavy episode drinking and stress level, khat chewing and tobacco use.

## Conclusions

More than one in ten adults were heavy episodic drinkers in the study area. This showed that heavy episodic drinking attracts attention to the urgent need to develop more effective prevention strategies. Occupation, economic status and altitude and substance use including tobacco and khat are factors associated with heavy episodic drinking among the adult population. Intervention strategies for reducing the prevalence of heavy episodic drinking need to address the population who chew khat, use tobacco and live in highlands. It is indicated that the impact of harmful lifestyle, including heavy episodic drinking, is disproportionately affect the deprived population [51, 52]. Intervention programs that aim to prevent heavy episodic drinkers should be designed appropriately for individuals from lower wealth status for reducing its significant negative impact.

## Data Availability

Raw data were obtained from study conducted by Arba Minch Health and Demographic Surveillance Site using WHO STEPS survey. Derived data supporting the findings of this study are available from the corresponding author upon request.

## Abbreviations

EDHS: Ethiopia Demographic and Health Survey
HDSS: Health and Demographic Surveillance Site
NCDs: non-communicable diseases
STEPS: STEPwise approach to Surveillance
WHO: World Health Organization
